# Plasma levels of soluble programmed death ligand-1 may be associated with overall survival in nonsmall cell lung cancer patients receiving thoracic radiotherapy

**DOI:** 10.1097/MD.0000000000006102

**Published:** 2017-02-17

**Authors:** Jing Zhao, Peng Zhang, Jianhua Wang, Qingsong Xi, Xueqi Zhao, Minghua Ji, Guangyuan Hu

**Affiliations:** a Department of Oncology, Tongji Hospital, Tongji Medical College, Huazhong University of Science and Technology, Wuhan; b Department of Radiation Oncology, Jiangsu Cancer Hospital, Nanjing, China.

**Keywords:** nonsmall-cell lung cancer, overall survival, PD-L1, thoracic radiotherapy

## Abstract

Immune-checkpoint signaling plays an important role in immunosuppression of tumors. We aimed to investigate the association of soluble programmed death-ligand 1 (sPD-L1) level in plasma with overall survival (OS) in locally advanced or inoperable nonsmall-cell lung cancer (NSCLC) patients treated with thoracic radiotherapy (TRT). We used ELISA to evaluate the sPD-L1 levels at diagnosis and during TRT in 126 clinically inoperable NSCLC patients. OS rates were followed up and recorded. SPSS software and GraphPad Prism 5 were used for statistics. In this study, the average sPD-L1 levels at baseline, week 2, and week 4 during TRT and post-TRT were 107.2, 51.3, 65.4, and 111.1 pg/mL, respectively. Levels of sPD-L1 at week 2 and week 4 were significantly less than at baseline, with both *P* values < 0.001. Using 96.5 pg/mL as the cutoff, patients with lower baseline sPD-L1 level had longer OS than those with higher sPD-L1 level (27.8 months vs 15.5 months, *P* = 0.005). Using multivariate analysis, the following factors were significantly associated with longer OS: female, adenocarcinoma, higher TRT dose, and lower baseline sPD-L1 level. Patients with both characteristics of lower baseline sPD-L1 level and higher TRT dose (BED10 ≥84 Gy) had the longest OS. To conclude, the lower baseline sPD-L1 level was significantly associated with longer OS in NSCLC patients treated with TRT, which may serve as an independent biomarker and needs further clinical study.

## Abstract 2

Supplemental Digital Content is available in the text

## 1 Introduction

Radiation therapy (RT) is the mainstay treatment for nonsmall-cell lung cancer (NSCLC) patients. Based on current knowledge, a higher radiation dose and concurrent chemoradiotherapy may improve the survival of patients, as has been demonstrated in previous trials.^[1,2]^ The accumulated evidence has recently shown that RT combined with immunotherapy, such as PD-1/PD-L1 blockade, could be a promising treatment strategy.^[3,4]^ Irradiation increases tumor destruction and triggers immune infiltration into tumors. In Zeng animal study^[5]^ involving mice with glioblastoma multiforme, improved survival was demonstrated with a combination of anti-PD-1 therapy and stereotactic body radiation therapy, compared with either modality alone.

Programmed death 1 (PD-1) is a transmembrane surface glycoprotein encoded by the CD274 gene located on chromosome 9. As the main ligand for PD-1, PD-L1 induces a coinhibitory signal in activated T-cells and promotes T-cell apoptosis, anergy, and functional exhaustion.^[6,7]^ There is evidence that tumor cells can express PD-L1 on the cell membrane by activated T-cells.^[8,9]^ This has been investigated in metastatic renal cell carcinoma (RCC), suggesting that primary RCC tumors with PD-L1 positivity—either on tumor cell membranes or inflammatory cells—will have a better response to PD-1/PD-L1-targeting therapies.^[10]^

For immunotherapies, many candidate biomarkers such as IFN-γ and TGF-β are under investigation.^[11]^ From the ASCO 2015 Annual Meeting, treatments targeting PD-1/PD-L1 pathway for NSCLC patients were widely reported. According to Tiffany's study,^[12]^ PD-L1 overexpression was significantly associated with increased CD8+ TILs and KRAS mutations in resected lung adenocarcinomas. It was reported that the PD-1 blockade demonstrated durable manageability as a first-line therapy for PD-L1+ metastatic NSCLC.^[13,14]^ Moreover, PD-L1 level in cell supernatants and staining slides could be used as a predictive factor of survival.^[15–18]^ A survival model established by Jiang et al^[18]^ showed that PD-L1 expression was predictive to OS in patients with squamous NSCLC. However, some other studies did not found such significant correlations.^[19,20]^

Soluble PD-L1 (sPD-L1) was easily detectable in human plasma using a commercial ELISA kit. Although the function and mechanism of release is debated, sPD-L1 could be alternative for clinical use, especially for patients without enough tumor tissue to test, which has been demonstrated by lots of previous studies.^[21–23]^ However, data were insufficient to describe the changes in the PD-1/PD-L1 level during thoracic radiotherapy (TRT). In this study, we aimed to investigate the changes of plasma sPD-L1 level in NSCLC patients receiving TRT, and to find the association between sPD-L1 level and overall survival (OS) in those patients.

## 2 Patients and methods

The procedures in this study were in accordance with the ethical standards of the responsible Committee on Human Experimentation of Tongji Hospital and with the Helsinki Declaration.

### 2.1 Eligibility

This is a prospective study (between 2009 and 2013). NSCLC Patients with locally advanced stage were eligible. Patient was required to have at least 1 measurable or assessable lesion, and prior TRT were not permitted. An Eastern Cooperative Oncology Group (ECOG) performance status of 0 to 2 was eligible. Initial evaluation consisted of a history and physical examination, ^18^FDG PET/CT or CT scan for staging, including full visualization of the liver and adrenal glands. A radionuclide bone scan and an MRI scan of the brain were required. Routine blood work, including complete blood count, and liver and renal function tests were also necessary. We obtained written informed consent from all patients before enrolling them onto the trials, in accordance with the procedures of the Institutional Review Board.

### 2.2 Treatment plan

Patients were treated definitively with TRT alone or with concurrent chemoradiotherapy. The gross tumor volume (GTV) was restricted to the primary tumor, any hilar or mediastinal lymph nodes with a short-axis diameter of at least 1 cm on CT, and any abnormal findings detected on bronchoscopy or mediastinoscopy. The clinical target volume (CTV) was routinely created by expanding the GTV by 0.5 cm. Clinically uninvolved hilar, mediastinal, and supraclavicular nodal regions were not purposely included in the CTV. The planning target volume (PTV) was defined as the CTV, plus 1.0 cm anterior, posterior, medial, and lateral margins and 1.0 to 2.0 cm superior and inferior margins, when necessary to account for respiratory motion.

The radiation dose was prescribed to isocenter and accounted for tissue heterogeneity. Radiation planning required that 100% of the PTV be encompassed by 95% of the isodose. Typically, a single-treatment plan was used, which consisted of a median of three noncoplanar static fields (range, 2–7). All patients received a daily treatment using 2.0 to 3.8 Gy fractions with beams ranging in energy from 6 to 25 MV.

Chemotherapy was administered weekly, concurrent with radiation on the same day each week. Carboplatin (AUC = 2) and Paclitaxel (45 mg/m^2^) were started on week 1 of TRT and continued weekly for 6 weeks.

### 2.3 Sample collections

Plasma samples were obtained from all eligible patients at diagnosis (baseline), 2 and 4 weeks after the first TRT treatment day (during TRT), and in 3 months of the last TRT treatment day (post-TRT). Plasma samples were collected into antiproteasic BD P100 blood-collection tubes (BD Diagnostics, Le Pont-De-Claix, France), and centrifuged and stored at −80°C.

### 2.4 Soluble PD-L1 measurement

Soluble PD-L1 in plasma was measured using an enzyme-linked immunosorbent assay (PDCD1LG1 ELISA kit, USCN Life Science, Wuhan, China), according to the manufacturer's instructions. The minimum detectable dose of sPD-L1 was 57 pg/mL. Each sample was analyzed in duplicate. Protein levels were calculated according to standard curves. The intraassay and interassay coefficients of variation were below 20%. The same assay and procedure were used to measure sPD-L1 levels for all studied cohorts.

### 2.5 Follow up

Follow-up procedures included the patients first being examined at the end of therapy, then every 3 months for 2 years, then every 6 months for 2 years, and yearly thereafter. Patients underwent a CT scan of the chest at every visit. At the time of progression, restaging procedures included CTs of the chest and abdomen and an MRI of the brain.

### 2.6 Statistical consideration

The primary endpoint is OS, which was calculated from treatment initiation, and estimated using the Kaplan–Meier method. OS was defined as the interval between the date of treatment initiation and the date of death, or last follow-up for patients still alive. Age, gender, smoking status, ECOG performance status, histology, concurrent chemotherapy, and radiation dose were evaluated for their association with each survival endpoint in multivariate analysis. Results are presented as median and 95% confidence interval (CI), unless otherwise specified. Analyses were carried out using SPSS 18.0 and GraphPad Prism 5 software. All tests were 2-sided, and a *P*-value < 0.05 was considered statistically significant.

## 3 Results

Table 1 summarizes the characteristics of all the eligible patients. A total of 126 NSCLC patients with stage IIIB were included in this analysis. The median follow-up for surviving patients was 25.0 months. Most patients had good performance status (with ECOG performance status score 0–1) before therapy. High biological equivalent dose at α/β = 10 (BED10) ≥84 Gy was delivered to 65 (51.6%) patients, and concurrent chemoradiotherapy was delivered to 86 (68.3%) patients.

**Table 1:**
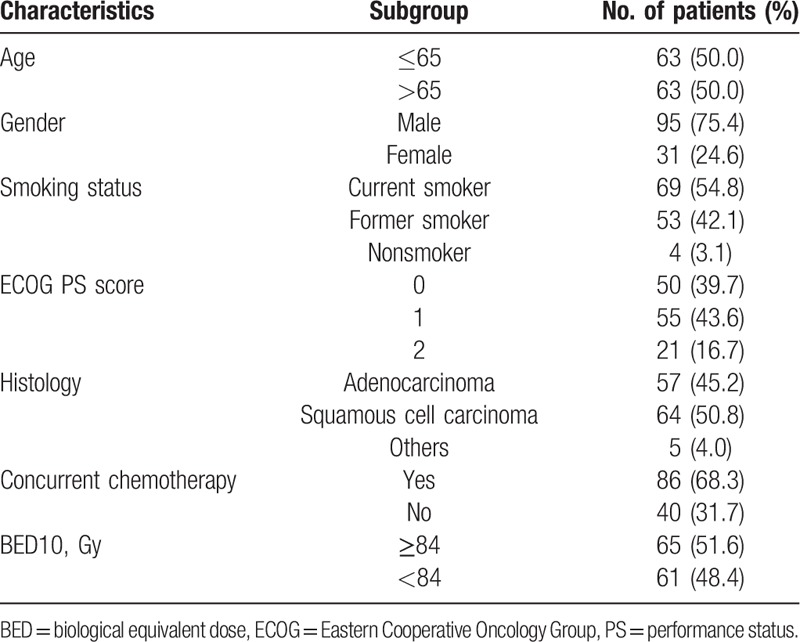
Baseline demographic and clinical characteristics of the patients at diagnosis.

### 3.1 Levels of sPD-L1 were decreased after TRT

**Figure 1:**
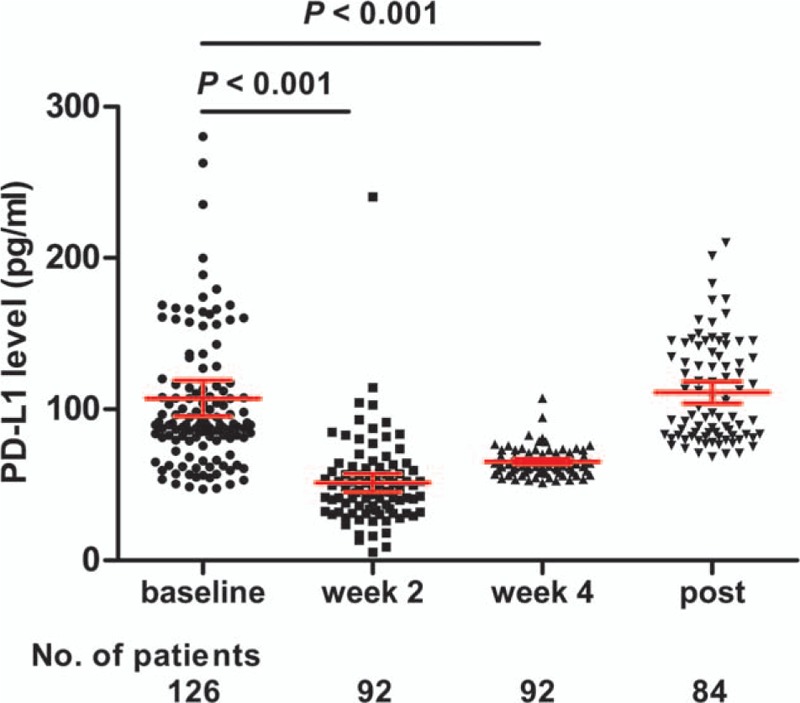
The changes of plasma soluble PD-L1 levels after thoracic radiotherapy. The bars show the values of mean ± 95% CI.

Levels of sPD-L1 varied significantly across the treatment period. The average levels at baseline, week 2, week 4, and post-TRT were 107.2, 51.3, 65.4, and 111.1 pg/mL, respectively. The sPD-L1 levels at week 2 were significantly lower than at baseline (*P* < 0.001), as were the levels of week 4 compared with the baseline (*P* < 0.001). However, there was no significant difference between the baseline and post-TRT levels (*P* = 0.62) (see Fig. 1).

### 3.2 High sPD-L1 level at baseline impacted OS

**Figure 2:**
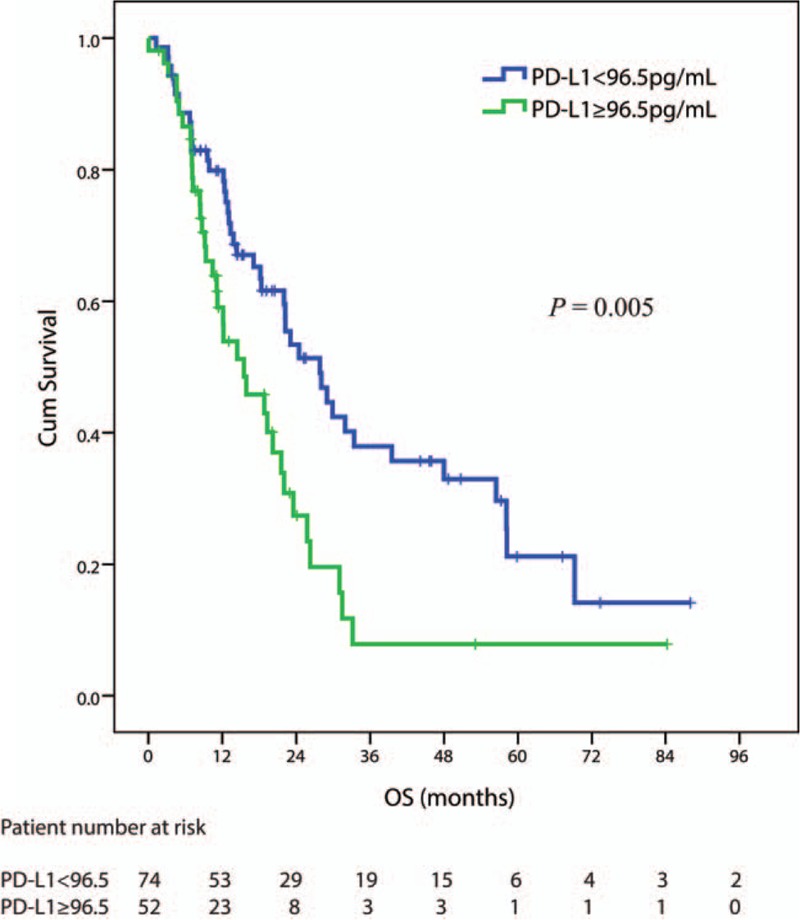
The correlation of plasma sPD-L1 level with the overall survival after thoracic radiation therapy. OS curves according to sPD-L1 levels at diagnosis for 126 patients considering the cut-off point at 96.5 pg/mL.

The optimal cutoff point for sPD-L1 measured at baseline was found to be equal to 96.5 pg/mL (MaxStat test *P* = 0.02, see Supplementary Fig. 1). The median OS (95% CI) in patients with lower sPD-L1 (<96.5 pg/mL) was 27.8 (19.7–36.0) months, compared with 15.5 (8.2–22.9) months in patients with higher sPD-L1 level (*P* = 0.005) (see Fig. 2).

**Figure 3:**
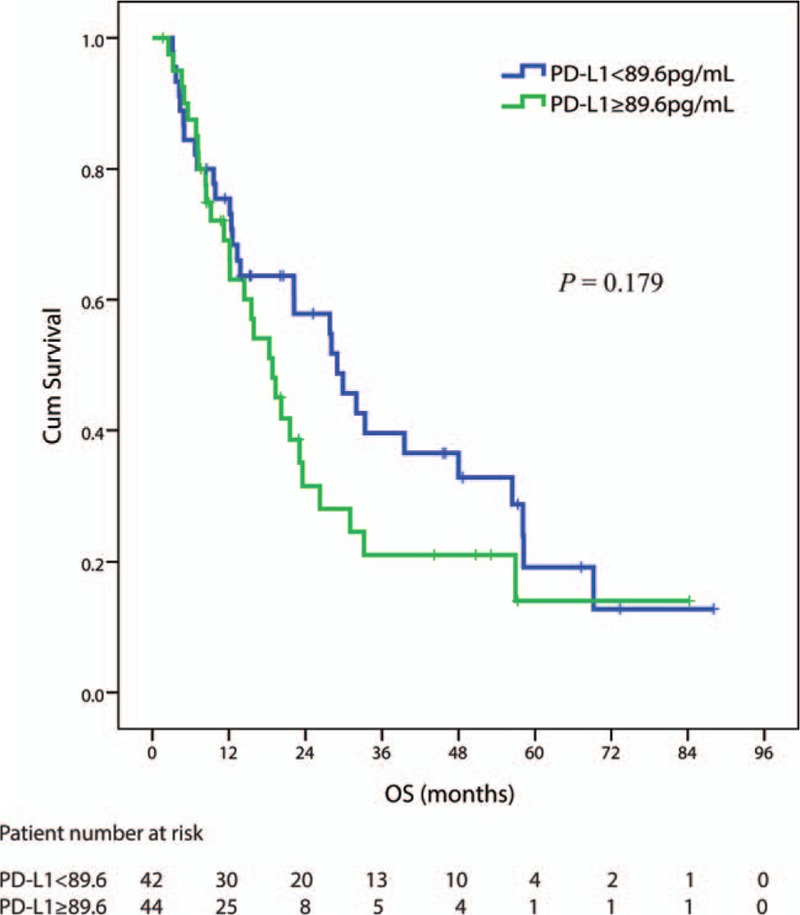
Kaplan–Meier estimates of OS in 86 patients receiving concurrent chemoradiotherapy considering the cut-off point for sPD-L1 at 89.6 pg/mL.

In 86 patients receiving concurrent chemoradiotherapy, the optimal cutoff point for baseline sPD-L1 was 89.6 pg/mL. In those patients, the baseline sPD-L1 level was not observed to be a significant factor with OS after treatment in those patients, although patients with lower levels had longer OS than those with higher levels. The median (95% CI) OS were 29.0 (18.8–39.1) and 18.8 (13.8–23.90) months, respectively (*P* = 0.179) (see Fig. 3).

**Figure 4:**
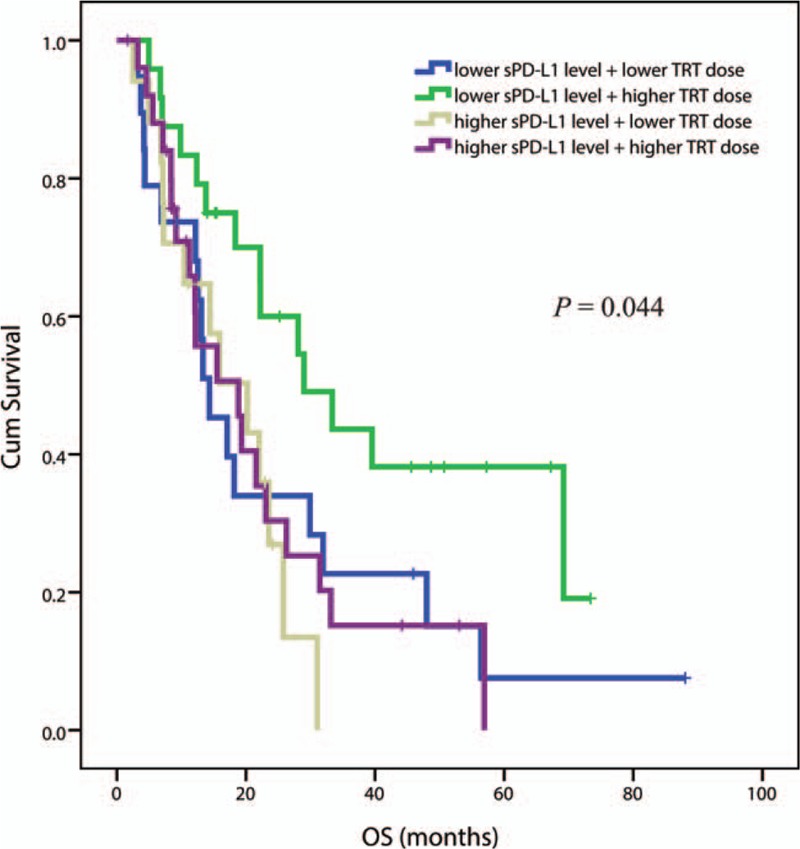
Kaplan–Meier estimates of OS in 126 patients with different status of risk factors of baseline sPD-L1 level and TRT dose. Patients were divided into 4 subgroups. TRT = thoracic radiation.

Considering clinical and blood characteristics, the only factors remaining significant after adjustment with *P*-value < 0.05 in multivariate analysis were: gender, histology, TRT dose, and baseline sPD-L1 level. The factors of female, adenocarcinoma, higher RT dose, and lower baseline sPD-L1 level were significantly associated with longer OS (see Table 2). Two different parameters were evidenced as independent prognostic factors for OS in this series, notably covariations of TRT dose and baseline sPD-L1 levels. The median (95% CI) OS in the subgroup with lower baseline sPD-L1 level and higher TRT dose was 29.0 (14.2–43.7) months, which was longer than any of the other 3 subgroups, which were 14.3 (8.9–19.7), 20.2 (10.2–30.2), and 18.8 (8.8–28.8) months for lower level + lower dose, higher level + lower dose, and higher level + higher dose, respectively (*P* = 0.044) (see Fig. 4).

**Table 2:**
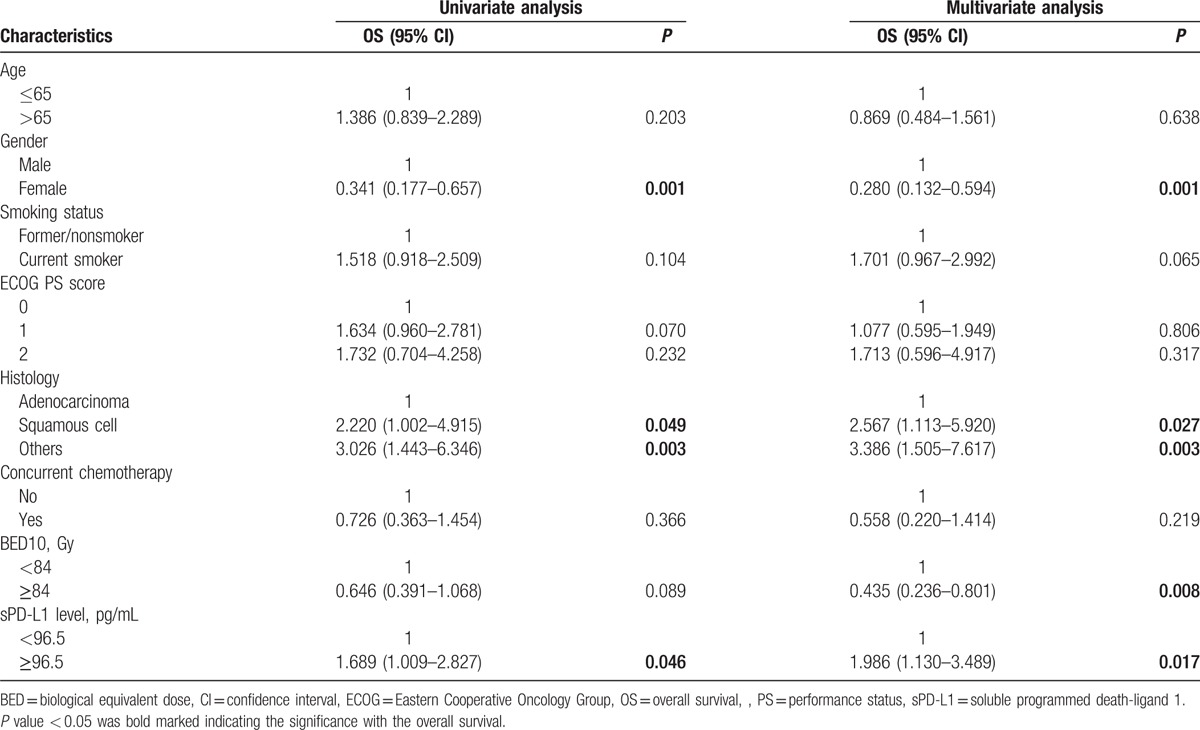
Results of the univariate and multivariate Cox analyses for OS after thoracic radiotherapy.

## 4 Discussion

This study supplied a novel blood marker for locally advanced NSCLC patients. The lower level of baseline sPD-L1 was significantly correlated with longer OS in NSCLC patients receiving TRT. Using the model of combined factors including both baseline sPD-L1 level and TRT dose could significantly predict OS. To our knowledge, this is the first clinical report with sPD-L1 in NSCLC patients treated with RT.

The upregulation of PD-L1 is a common phenomenon in cancer patients,^[24]^ leading to enhanced T-cell immunodeficiency and high immune suppression.^[25]^ PD-L1 could possibly be a consequence of proinflammatory cytokine produced by tumor-infiltrating lymphocytes (TILs). For example, IFN-γ produced by inflammatory cells could act as a potent PD-L1 upregulator.^[26]^ This is thought to be important for coinhibition during the T-cell initiation of an immune response. CD8+ T-cells play a major role in cellular responses, including in antitumor immune defense, while TILs contribute to good clinical outcomes in many types of cancer.^[27]^ Muenst et al^[28]^ found expression of PD-L1 was significantly associated with an increase in the number of TILs in breast cancer. It might be an indication that as the tumor progresses, PD-L1 is increasingly produced by tumor cells in order to evade the mounting antitumor immune response. Ghebeh et al^[29]^ also found PD-L1 expression by TILs in 41% of their specimens, predominantly on CD4+ T-cells. Moreover, expression of PD-L1 on TILs might inhibit T-cell function by binding to PD-1 expressed on other TILs, as demonstrated by Seo et al.^[30]^ The capacity of the PD-1/PD-L1 interaction to downregulate a CD3/CD28-stimulated response shows that PD-1 engagement results in the delivery of a strong inhibitory signal.^[31]^

The mechanisms by which elevated sPD-L1 levels contribute to poor prognosis are not clear. PD-1 was observed to induce drug resistance via the AKT signaling pathway in myeloma cells.^[32]^ Additionally, the PI3K/AKT inhibitors could reduce the PD-1/PD-L1's induced resistance to antitumor agents.^[32]^ The clinical data have shown that PD-L1 is important for immune evasion by tumor cells. It has been reported that a doubling of sPD-L1 levels was associated with a 41% increased risk of death (*P* = 0.01).^[22]^ Azuma et al^[33]^ reported that high expression of sPD-L1 was associated with the presence of epidermal growth factor receptor (EGFR) mutations in surgically resected NSCLC and was an independent negative prognostic factor for this disease. In Rossille's study,^[21]^ elevated sPD-L1 was correlated with a poorer prognosis for patients with B-cell lymphoma treated with R-CHOP chemotherapy (*P* < 0.001), using a cutoff of 1.52 ng/mL for PD-L1 level. ASCO 2015 Annual Meeting reported that NSCLC patients with PD-L1-negative tumors showed longer progression-free and OS than those with PD-L1-positive (median PFS, 17 months vs 11 months; *P* = 0.31, median OS, 52 months vs 25 months; *P* = 0.38; respectively).^[34]^ Increasing data indicate that the PD-1/PD-L1 signaling pathway is related to immune suppression and disease progression, such as melanoma, renal, urothelial, gastric, lung, and colorectal cancer.^[35–39]^ Further studies are needed to identify how PD1/PD-L1 signaling impacts prognosis.

A high TRT dose has been observed to be a significant predictive factor for OS. Investigators from Memorial Sloan-Kettering Cancer Center reported that patients who received ≥64 Gy had better survival than those who received <60 Gy in stage III patients receiving chemoradiotherapy.^[40]^ Kong et al^[2]^ have reported the dose effects of 237 patients with stage III NSCLC, showing that BED was a significant prognostic factor associated with the risk of death (HR = 0.96 for each Gy, 95% CI: 0.95–0.97, *P* < 0.001). In a randomized trial from China, stage III patients treated with a total dose of 68 to 74 Gy had significantly better 5-year tumor local control and 2-year OS than those treated with 60 to 64 Gy (51% vs 36%, *P* = 0.032; 39.4% vs 25.6%, *P* = 0.048).^[41]^ A high radiation dose was considered to induce an anti-tumor immune function including increasing inflammatory cells and effective T-lymphocyte invasion. However, it is usually difficult to clarify the relationship between radiation and the immune effect, which may largely depend on the biological equivalent radiation dose as well as immune cell types.^[42,43]^ Preclinical studies have indicated the critical role of radiation with appropriate dose and fractions inducing tumor immunogenic antigen release during antitumor immune response, in which new peptides were generated in complex antigenic environment. For example in Sharabi et al's study^[44]^ using B16-OVA melanoma cells and 4T1-HA breast carcinoma cells, it was observed that radiation could enhance tumor-specific antigen-MHC complexes, increase T-cell infiltration into tumor cells, and upregulate antigen cross-presentation in the draining lymph nodes. These results are expected to be demonstrated in future clinical trials.

There are some limitations to this study. Firstly, we only test the soluble PD-L1 level in the blood and we observed a decreased trend in plasma level during TRT, which was opposite to the results in mice.^[45,46]^ As far as we know, TRT together with concurrent chemotherapy in this study induced tumor deaths in most of the patients, which contributed to the low PD-L1 level. However, whether the change of PD-L1 expression on tumors affected the level of soluble PD-L1 in plasma is unclear up to now. Secondly, it is interesting that the PD-L1 level recovered to the baseline after RT, which may be associated with the increase in inflammation; however, no such data have been achieved. In Deng et al's animal study,^[45]^ the synergistical effects of TRT and anti-PD-L1 treatment were first observed, which encourage us to perform more clinical trials in the future.

To conclude, the baseline plasma sPD-L1 level before TRT could be an independent prognostic biomarker for NSCLC patients. Patients with lower baseline sPD-L1 level together with the treatment of high TRT dose (BED10 ≥84 Gy) were significantly correlated with longer OS.

## Supplementary Material

Supplemental Digital Content
