# Supplementary material for: Plasma levels of soluble programmed death ligand-1 may be associated with overall survival in nonsmall cell lung cancer patients receiving thoracic radiotherapy
Source: Medicine (Baltimore). 2017 Feb 17;96(7):e6102. doi: 10.1097/MD.0000000000006102 (PMC5319514; doi:10.1097/MD.0000000000006102)

**Supplementary Fig. 1.** Standardized log-rank statistic versus plasma sPD-L1 cutoff point. The optimal plasma sPD-L1 threshold is determined for the maximum log-rank statistic.


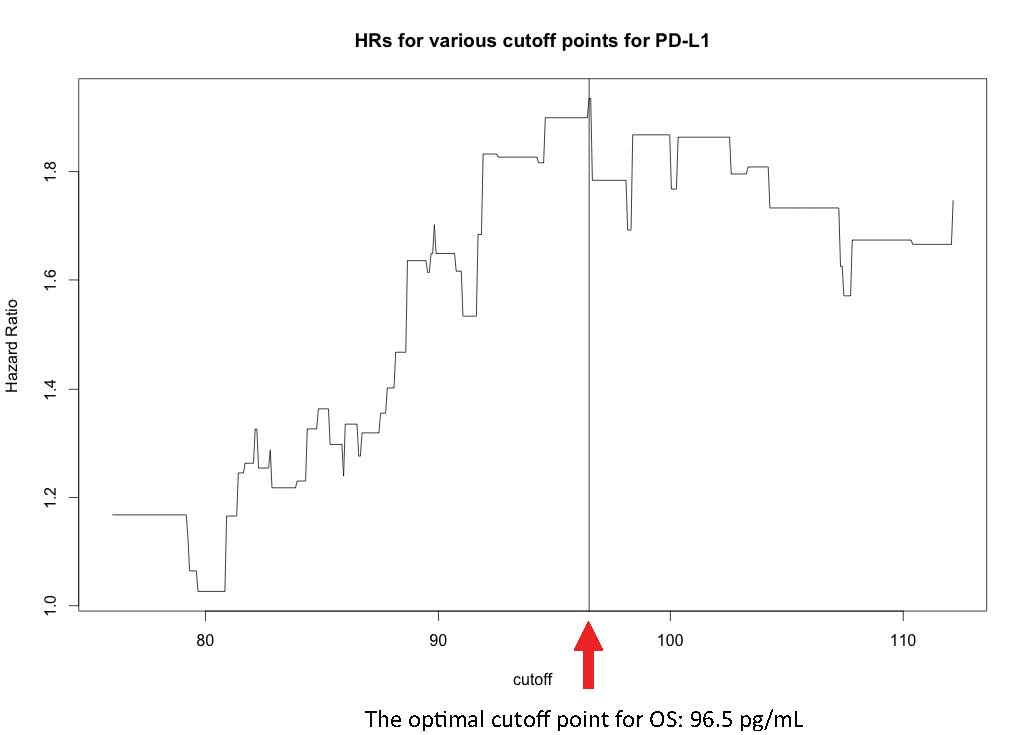

Supplement: Supplemental Digital Content [file medi-96-e6102-s001.doc]
